# How Psychological Wellbeing and Digital Mental Health Services Intervene the Role of Self-Efficacy and Affective Commitment of University Students in Their Performance?

**DOI:** 10.3389/fpsyt.2022.946793

**Published:** 2022-07-07

**Authors:** Wang Min, Gao Jun, Liu Feng

**Affiliations:** ^1^School of Marxism, Zhejiang A&F University, Hangzhou, China; ^2^Heilongjiang Vocational College of Art, Harbin, China

**Keywords:** self-efficacy, affective commitment, psychological wellbeing, digital mental health service, students' performance

## Abstract

Student performance is a critical factor in academic achievement. Other factors like the students' self-efficacy, affective commitment, and psychological wellbeing play a significant role in shaping their performance. The present study aims to understand the role of self-efficacy, affective commitment, and psychological wellbeing in the students' performance. To carry out the study, the data were collected from the 308 students currently enrolled in the public sector universities of China. Smart-PLS is used to check the validation of the proposed hypotheses. Partial least square structural equation modeling is used for hypothesis testing. Results of the study show that self-efficacy does not play a role in the student performance in public sector universities; however, the affective commitment of the students plays a significant role in their performance. The psychological wellbeing of the students has a substantial influence on their performance. Furthermore, the results have also indicated that psychological wellbeing is an important indicator of student performance. It has also been revealed that psychological wellbeing significantly mediates the relationship between self-efficacy, affective commitment, and student performance. The students who availed of the digital mental health services were found to have a low relationship between their self-efficacy and performance.

## Introduction

Students' performance is closely connected to the socio-economic development of a country. Students' performance is critical in developing the top-quality graduates who may become leaders and workforce in a particular region. Therefore, they are accountable for the country's social and economic progress. The students' performance has gotten a lot of attention in previous studies. Behavioral, economic, societal, interpersonal, and environmental factors influence student performance. These elements have a significant impact on student achievement. They range from region to region and individual to individual. Mushtaq and Khan (1) indicated that earlier studies emphasized determinants of students' performance, such as teacher capability, classroom atmosphere, gender bias, style of teaching, and household education level.

Most researchers throughout the world have used grade point avaerge (GPA) to evaluate student performance. Researchers use GPA to assess students' performance over a semester. Some researchers have used GPA to measure student performance. Some studies evaluated students' performance based on the previous year's results or the result of a specific course. Various studies have attempted test results or have previously considered performance for a certain subject (2). Student performance has been an important aspect of higher education at various institutions. Some scholars consider it the most crucial one when evaluating the institution (3). According to Maldonado and Salanova (4), student performance is the most relevant attribute of evaluating university setups. Following this logic, there is a connection between higher education institutions and commercial organizations. It is because both are concerned with the standard of performance of their employees and how to preserve and improve that performance (4). If students consider their performance responsibilities, it becomes critical to discover individual and group aspects that may impact their success (5). Further research is required to produce evidence-based treatments to increase students' learning and performance (4).

Students' performance is shaped by many factors, including self-efficacy (SE) and affective commitment at the academic level. According to Bandura (6), the Social Cognitive Theory includes SE as a major personal characteristic. It is described as a person's conviction in his ability to plan and carry out actions to attain the intended outcomes (6). This concept has received a lot of attention from academic scholars. Prior research has shown that it is a powerful predictor of performance (7). SE correlates with students' educational success across academic subjects and degrees (4). Although many evidence supports the direct impacts of SE beliefs on students' performance, few studies have looked at the psychological wellbeing that mediates the SE–performance link. Such research is vital to explore how SE influences students' performance. It may help create instructional actions and programs to increase student performance (8).

Self-efficacy is typically articulated in academic contexts from the perspective of academic SE. It describes student perceptions of their capacity to achieve learning objectives. A lot of research shows how vital this type of SE is for education and future achievement. Previously, it has been investigated in various settings, encompassing early childhood, middle school, and higher education institutions (9). Integrative research has revealed complicated correlations among a variety of factors. These studies suggest a process through which SE affects students' performance. This kind of performance is controlled and mediated by a variety of factors. The existing evidence strongly supports the association between SE and student performance. Nevertheless, the literature on interactions and mechanisms between these is more complicated.

This is possible due to the absence of internal consistency in the models evaluated with different combinations of exploratory variables. Despite the availability of relevant literature on the connection between SE and student performance, no research has examined the impact of SE on student performance in a higher education environment (9). This research is designed to fill this gap based on psychological wellbeing as a mediator and digital mental health services as a moderator. The other determinant of students' performance in the current research is an affective commitment, which is associated with it. Institutions are putting in a lot of effort to improve the student experience to increase student engagement, performance, and enrollment.

Institutions have a vested interest in producing a devoted student body, yet commitment is an essential part of student performance. Students are required to study at an institution wherever they wish to spend the next 4 years of their lives. Disengaged students may detract from group participation, create havoc, and spread bad information. Therefore, affective commitment is a crucial contributor to students' performance. It encompasses individuals' continual desire to feel attached to their place of study (10).

Affective commitment seems to have a role in people's success and job satisfaction. This is not always present in relationships with other variables like trust. In the higher education context, Bowden and Wood (11) found an affective commitment to become a key motivator of trust. Institutions keep students who are emotionally invested in them. The study addresses the dilemma that higher education institutions confront in figuring out how to effectively develop long-term, committed relationships between students. This approach can help fight the emotional and financial strains of higher education (10). The suggested concepts in this study attempt to add to student performance in this way. Previously, no study has ever looked into the relationship of affective commitment of students with their performance. Therefore, this study fills the gap by finding the impact of affective commitment on students' performance.

This study fills the gaps in previous studies about the linkage of SE with students' performance by evaluating psychological wellbeing as a mediator. Factors, such as personality, self-progress, and meaningful participation describe individuals' psychological wellbeing (PsWB) (12). Researchers claim that PsWB is a useful indication of health beyond mental wellbeing. Furthermore, PsWB is crucial in personal and societal development (12). People who prioritize psychological health and wellbeing are better equipped to respond to situations as they emerge and find appropriate solutions. Many researchers have considered the importance of numerous contributing elements in PsWB. Wellbeing is a product of environmental circumstances and a person's degree of self-determination (11).

How PsWB affects students' performance is less known. According to research, students' wellbeing and health are the most important elements influencing their academic success and performance. PsWB has been linked to various human and organizational outcomes, including improved performance and effectiveness, client satisfaction, work engagement, and corporate citizenship behavior (13). The success and productivity of educational institutions are linked to the performance of their students and employees. Most of the research has demonstrated the importance of students' performance in terms of quantifiable activities, behaviors, and results. Such behaviors help achieve institutional goals and prompt scholars to investigate what motivates students to perform well (13).

Apart from PsWB, digital mental health services are gaining momentum. These services are directed toward providing the students with activities that help in improving mental health digitally. Recently, it has been noted that learning shifted toward online education due to a recent pandemic. In the recent decade, it has been assessed that college-level students got symptoms of depression and anxiety that were not prevalent earlier (14). Therefore, digital health services also got a chance to function in these difficult times. These services help reduce anxiety and depression among those who stayed mostly at home during these times. It is also evident from current times that digital health services positively impact the PsWB of people, including students (15).

Therefore, current research considers that digital mental health services could have a moderating effect on students' performance. This research addresses some questions, including RQ1. What impact may SE have on students' performance? RQ2. How does the effective commitment of students to their institutions impact their performance? RQ3. What role can PsWB as a mediator play between SE, affective commitment, and students' performance? RQ4. How can digital health services moderate the relationship of SE with students' performance? To answer all these questions, current research evaluated the direct impacts of SE and the affective commitment of students to their institutions on their performance. This study also looked into the indirect and mediating role of PsWB between SE, affective commitment, and students' performance. The moderating role of digital mental health services was also tested in this research.

## Theoretical Support

This research is based on Social Cognitive Theory. It was first presented by Bandura (16). He was among the first to look into the role of SE in driving people's behaviors. This theory is among several other theories which were meant to be associated with the processes helpful in regulating behaviors. Due to its significance, this theory got enormous attention in the last few years. This theory proposes that behavior is motivated and regulated by physical, social systems, and interior self-influence elements. SE is a crucial component of these self-influence elements (16). It relates to a person's assessment of their own ability to develop and implement the actions necessary to attain a desirable outcome. SE has been investigated in a variety of psychological fields, including cessation of smoking, eating behavioral therapy, dependency recurrence, job behavior, athletic ability, and performance of students (9).

This study is also supported by the framework of self-regulated learning by Printrich (17). SE occurs as a crucial motivating component within an integrated framework for self-regulated learning and serves as a core mechanism to interpret the self-monitoring mechanisms defined by Social Cognitive Theory (16). This theory describes how social, environmental, psychological, and intellectual elements interact to determine student performance outputs like Grade Point averages, test scores, and students' final grades. Using a range of complicated data modeling and mediation methodologies, the relationship between SE and the range of factors within this framework for determining students' performance in academic environments has been thoroughly examined (9, 18).

Affective commitment is a kind of commitment that was reported at the organizational level. It is used as a behavioral aspect of students with their institutions. It is supported by the sit-bet theory given by (17). PsWB is utilized as a mediator in this research and is supported by the self-determination theory. It is described as a person's right to make choices and exercise control over the situation to improve their mental wellbeing and PsWB (19). This theory divides psychological requirements into autonomy, connectedness, and competency. Such cognitive demands are considered necessary for a person's pleasure and fulfillment. Authors argue that people who are proud and satisfied in their activities are more dedicated to their organizations based on this principle.

### Self-Efficacy and Student Performance

One of the most essential aspects of impacting students' performance is SE. Students' SE relates to perceptions and beliefs about their capacities to succeed academically. It also relates to their confidence in their capacity to complete academic assignments and understand the contents successfully (20). Students with high SE beliefs get exceptional results by enhancing their dedication, effort, and persistence. Students who have high SE relate their shortcomings to a lack of effort instead of a limited ability, whereas those who have poor SE ascribe their shortcomings to a lack of ability (21). SE can affect the selection of tasks, as well as persistence in performing the task. To put it another way, students with poor SE are more inclined to avoid, post-pone, and abandon their assignments (20, 22).

Someone with a high degree of SE is more dependent on himself when challenged with complicated problems to find a solution. Such a person also shows patience during the endeavor. He tries to put in more effort and struggles harder to face adversity (24, 25). As a result, SE appears to be one of the biggest essential determinants of students' performance (11). A research of more than 200 university students by Alyami (26) found that SE had a favorable and substantial impact on students' performance. SE has been proven in other research to have a significant impact on students' education, ambition, and educational achievement (8).

Furthermore, research has looked into the impact of SE on students' performance in a variety of contexts, including SE for accomplishing particular topic functions like geometry or algebra problems, SE for superior performance and academic achievement of a specific position in a course, and SE for overall success in a degree program (9). SE has continuously been proven to relate positively with students' performance regardless of the educational context in which it is tested. This is not always true as all efficacious students do not have the tendency to excel in their performance. This perception is drawn from a manuscript that states that practice is also required to achieve good and sustainable performance (23). Based on this supposition, it can't be claimed right away that students' SE will always impact the students' performance. If students lack SE, then it will also lead to compromised performance. Therefore, in the current context of research, it is assumed that it might have an impact on students' overall performance. Similarly, it is also assumed that SE may not impact the students' performance alone. So, the authors suggested a possible role of SE in shaping the students' performance. Hence, the following hypothesis was built in this regard.

***H***_**1**_***:*** *Students' performance is influenced by the impact of SE*

### Affective Commitment and Students' Performance

In the early 1960s, the sit-bet theory pioneered the notion of organizational commitment (27). Workers' cognitive relationship to the organization and engagement is characterized as organizational commitment. It is characterized as the conviction of individuals in their organizational standards. It may also be described as an employee's devotion to the organization. It is also an employee's willingness to engage in organizational activities. There are three types of commitment, affective, procedural, and continuance commitments, which are linked yet separate. Students with affective commitment are emotionally invested in their institutions (28, 29). People with normative commitment remain dedicated to their organizations because they feel obligated to serve. Professionals that are devoted to their organization continuously do this because the costs of quitting are prohibitive (30).

Affective commitment is demonstrated to have the greatest impact on organizational results of all the characteristics of organizational commitment (13). It is a stronger predictor of citizen behavior, job performance, and low turnover intentions (30). Workers with stronger affective commitment showed that they perform better than those with a low feeling of responsibility and dedication for their firm (9). Research in Denmark studied healthcare workers and discovered that employees' affective commitment is linked to various performance attributes. Employees' affective commitment was also found to be highly associated with work performance among various individual and organizational outcomes (31).

Because of its relevance as an effective driver of performance outcomes, such as low turnover, work satisfaction, and job performance, the construct of affective commitment has been employed as a predictor variable, mediator, and moderator variable in much research (13). Previously it has been studied as a predictor of employees' job performance; therefore, based on this analogy, it is assumed in current research that it may also impact students' performance. Students' performance could be the outcome of students' affective commitment to their institutions. Hence, it was suggested in current research that the affective commitment of students is the determinant of their performance. So, the following hypothesis was developed.

***H***_**2**_***:*** *Affective commitment has an impact on student performance*

### Psychological Wellbeing

People's valued experiences help them become more productive in their job and are referred to as wellbeing. It is a subjective term that characterizes people's pleasure, desire, fulfillment, contentment, capacities, and job successes. Hedonic and eudaimonic wellbeing are the two categories of wellbeing (32). The scales used to evaluate the wellbeing of employees are divided into two categories. These categories include wellbeing at a subjective and personal level (13). Hedonic wellbeing is subjective, and eudaimonic wellbeing is personal (33). The cognitive component of hedonic wellbeing refers to people's conscious judgment of all elements of their lives.

Hedonic well-affective being's component refers to people's feelings resulting from experiencing pleasant or bad emotions in response to life (33). The other type, Eudaimonic wellbeing, describes people's innate characteristics and achievement of their full potential. This type of wellbeing is defined as a joyful existence based on self-reliance and personality (33). According to Pavot and Diener (34), hedonic wellbeing focuses on enjoyment, pleasure, and good emotions and has a higher positive effect and better life satisfaction. Eudaimonic wellbeing, on the other hand, differs from hedonic wellbeing in that it emphasizes actual self- and personal growth and awareness of one's best capacity and mastery (34). Hedonic and eudaimonic wellbeing have been discovered to be somewhat associated in the past, but they are two separate types (35).

Prior studies have quantified PsWB using a variety of measures, including employee satisfaction, support networks, and overall physiological and psychological health. PsWB has been linked to hedonic and eudaimonic wellbeing in a few studies, but more research is needed (36, 37). Therefore, this study evaluates PsWB using two validated measures: hedonic wellbeing, which refers to people's overall pleasure with life, and eudaimonic wellbeing, which refers to people's emotions of personal success. Organizational studies have paid some attention to the wellbeing of employees. The previous study has shown that healthier and happier employees work more, perform much better, and produce more (13). Employee wellbeing also positively impacts their work-related behavior and attitudes, such as enhancing citizen behavior, improving job performance, and reducing job conflict and absence.

There is an indication that wellbeing promotes job attitudes, but the relationships between PsWB, SE, and affective commitment are less understood (38). Furthermore, previous research has shown that an affective commitment is either a determinant or a predicted wellbeing variable. On the other hand, affective commitment as a predictor variable of PsWB has received little attention from scholars. As a result, the authors want to investigate affective commitment as a contributing variable of PsWB in this study since students who are successful and happy in their lives seem more likely to be connected to their institutions (38). This literature support also predicts that PsWB may mediate the relationships between SE, affective commitment, and students' performance. Therefore, the following hypotheses were suggested.

***H***_**3**_***:*** *Psychological wellbeing has an impact on student performance*
***H***_**4**_***:*** *Psychological wellbeing helps in mediating the association of self-efficacy and student performance*
***H***_**5**_***:*** *Psychological wellbeing mediates the relationship between affective commitment and student performance*

### Digital Mental Health Service

Over the past few years, around 31% of college students worldwide positively identified with a mental health issue. It is becoming increasingly clear that getting help for these prevalent mental health issues is tough. Several students lack mental health awareness and therefore do not identify the need for therapy, believing that their anxiety and depression symptoms are just the result of college stress and hence do not require treatment. The students who realize the need for mental health services frequently experience various hurdles to seeking help, regard the care provided as burdensome, and are doubtful of its usefulness (39–41).

Counseling facilities on campuses were very good, and provided mental health services. Several counseling facilities around the country, on the other hand, are not well-funded. They face difficulties reaching the needy students and are overburdened most of the time. The digital mental health services, through Web and Mobile platforms, allow students with co-occurring disorders to receive therapy while avoiding many hurdles to traditional mental health care, such as stigma and time (42, 43). For general adult population, the evidence basis for digital mental health interventions is large, and the scientific basis for university and college students is fast growing (44, 45).

**Figure 1:**
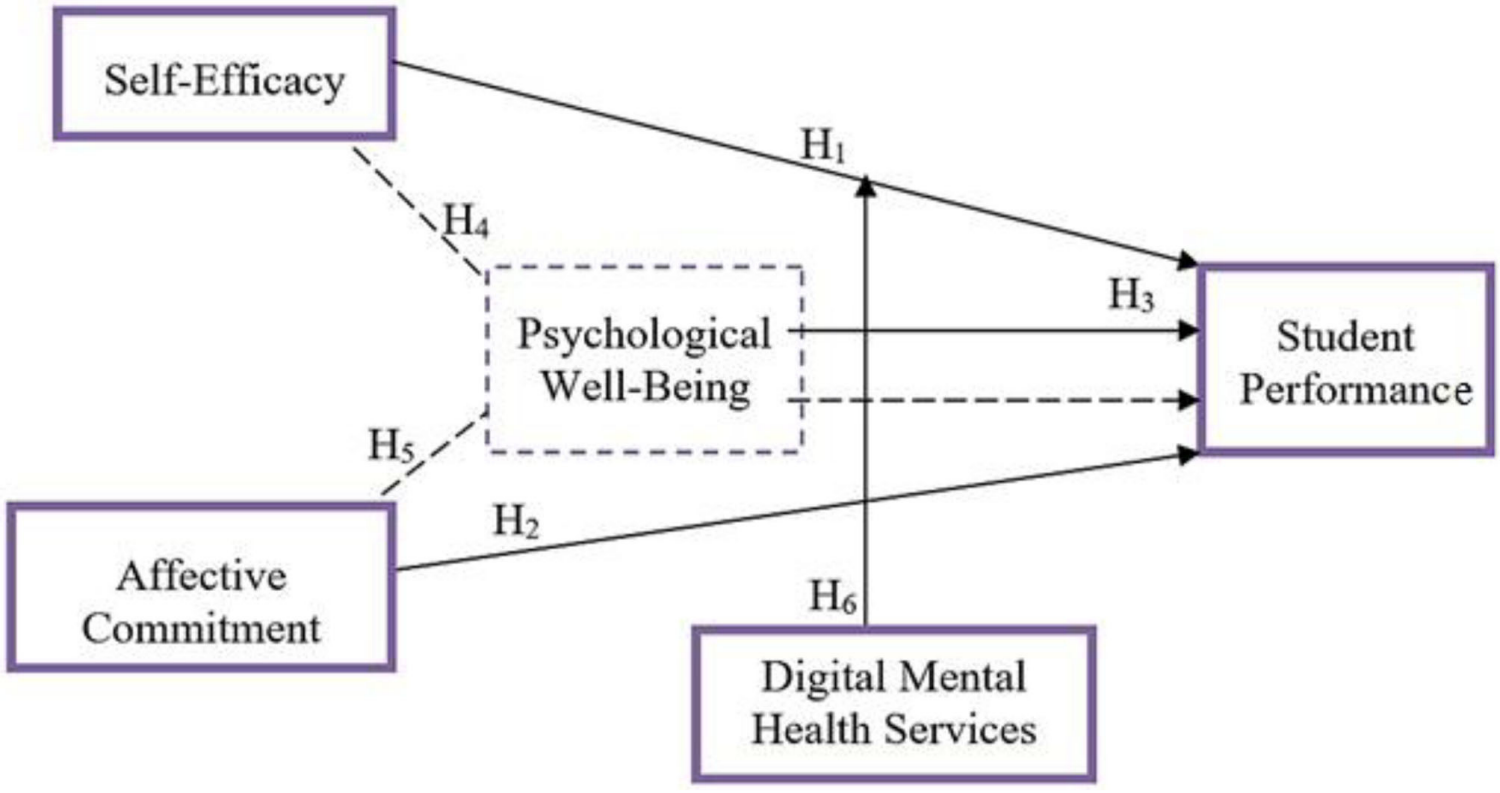
Conceptual framework.

A comprehensive review of digital therapies for mental health among students receiving higher education, published in 2013, indicated that such treatments have the potential to improve the symptoms of some mental health conditions, and additional testing is required. A comprehensive review of the delivery of these services through software and Websites for university students published in 2014 revealed that these interventions could help students with despair, stress, and anxiety. A comprehensive review and meta-analysis published in 2018 discovered that online therapies can have small-to-moderate benefits on various mental health disorders (46, 47). To tackle such mental health-related issues of students, which may influence their performance, digital health services are considered moderators in the current research. So, the following hypothesis is developed. The conceptual framework of the study developed based on the study theories and literature review is given in Figure 1.

***H***_**6**_***:*** *Digital mental health services moderate the relationship between self-efficacy and student performance*

The conceptual framework of the study is given in Figure 1.

## Methodology

The current study entails the deductive approach to examine the role of SE and affective commitment of the students in their performance with the mediating role of psychological wellbeing. This further investigates the moderating role of digital mental health services in the relationship between SE and student performance. Therefore, a quantitative research design has been followed to validate the study hypothesis. The hypotheses were developed to determine the impact of predictor variables (SE and affective commitment) on the outcome variable (student performance). This research design was beneficial in reducing the possible biases in the study (48).

The students studying in the public sector universities of China were the target population of the current study. Convenience sampling was deployed as the sampling technique for the study. This technique was used because it is less costly and requires less time to take data from a large number of respondents (49). Also, under this technique, the data is obtained from readily available respondents. The study was quantitative in nature; hence a self-administered survey was used to obtain quantitative data from the respondents for the study. The university administration had been approached to get permission to let the willing students participate in the survey.

The ethical protocol had been observed throughout the process. After getting approval from the administration, correspondence was made with the students in their free time slots in the class. They were first oriented on the purpose of the study and that the data obtained will solely be used for the research purpose. They were made to feel comfortable by not disclosing their identities. They were also told that they can mark the response that best fits their understanding as there is no right answer to the questions and that they will not be judged. A sample size of 430 was determined for the study. The current study's unit of analysis was the employees working in the public sector of China. The questionnaires had been distributed among the students and those who filled them right then were collected, while the rest of the questionnaires were collected a week later. After the data was collected from the employees, the data was then analyzed using the partial least square structural equation modeling. The software used in this study was Smart-PLS 3.3.9.

### Measurement Scale

The survey instrument for the present was a self-administered questionnaire. The questionnaire included the items for the variables of the study. The items were adapted from previous research carried out in the same context as the present study. The current study included five variables: SE, affective commitment, psychological wellbeing, student performance, and digital mental health service. Van Waeyenberg et al. (50) adopted a six-item scale for the affective commitment variable (affective commitment). A four-item scale for the variable student performance was also adopted from Van Waeyenberg et al. (50). A four-item scale for the variable psychological wellbeing was adopted from Darvishmotevali and Ali (51). An eight-item scale for the variable self-efficacy was adopted from the study by Tsai et al. (52). A five-item scale for the variable digital mental health service was adopted from the study by Marino-Francis and Worrall-Davies, (53) and had been modified according to the present study. The quantitative data was obtained using a five-point Likert scale against each of the items. The responses ranged from 1 to 5, where 1 = strongly disagree and 5 = strongly agree.

### Demographics Details

A total of 308 usable questionnaires were received from the respondents. Table 1 shows that, out of these 308 participants, 151 (49.02%) were male employees while female employees were 159 (51.62%). The students who had an age between 18 and 23 years were 165 (53.57%), the students who have an age between 24 and 28 years were 128 (41.55%), and the age of students above 28 years have 15 (4.8%). Moreover, 175 (56.81%) students have bachelor's degrees, 122 (39.61%) students have master's degrees, and 11 (3.57%) students have Ph.D.

**Table 1:** Demographics analysis.

**Demographics**	**Frequency**	**Percentage**
**Gender**		
Male	151	49.02%
Female	157	50.98%
**Age (years)**		
18–23	165	53.57%
24–28	128	41.55%
Above 28	15	4.8%
**Education**		
Bachelor	175	56.81%
Master	122	39.61%
Ph.D	11	3.57%

*N = 308*.

## Data Analysis and Results

Smart-PLS 3.3.9 was used for data analysis of the data acquired from the employees working in the public sector. This software helped to carry out the structural equation modeling (SEM) technique (54). This software analyses the data using two different stages. In the first stage, the measurement model is analyzed, which helps to conduct validity and reliability of the data. Data validity was carried out through the analysis of factor loadings, average variance extracted (AVE), HTMT, and Fornell and Larker Criteria. The reliability of the data in the measurement model was analyzed through the analysis of Cronbach alpha and composite reliabilities. In the second stage, the structural model was analyzed to confirm the proposed hypotheses by examining the sample means, *P*-values, standard deviation, and t-statistics.

### Measurement Model

**Figure 2:**
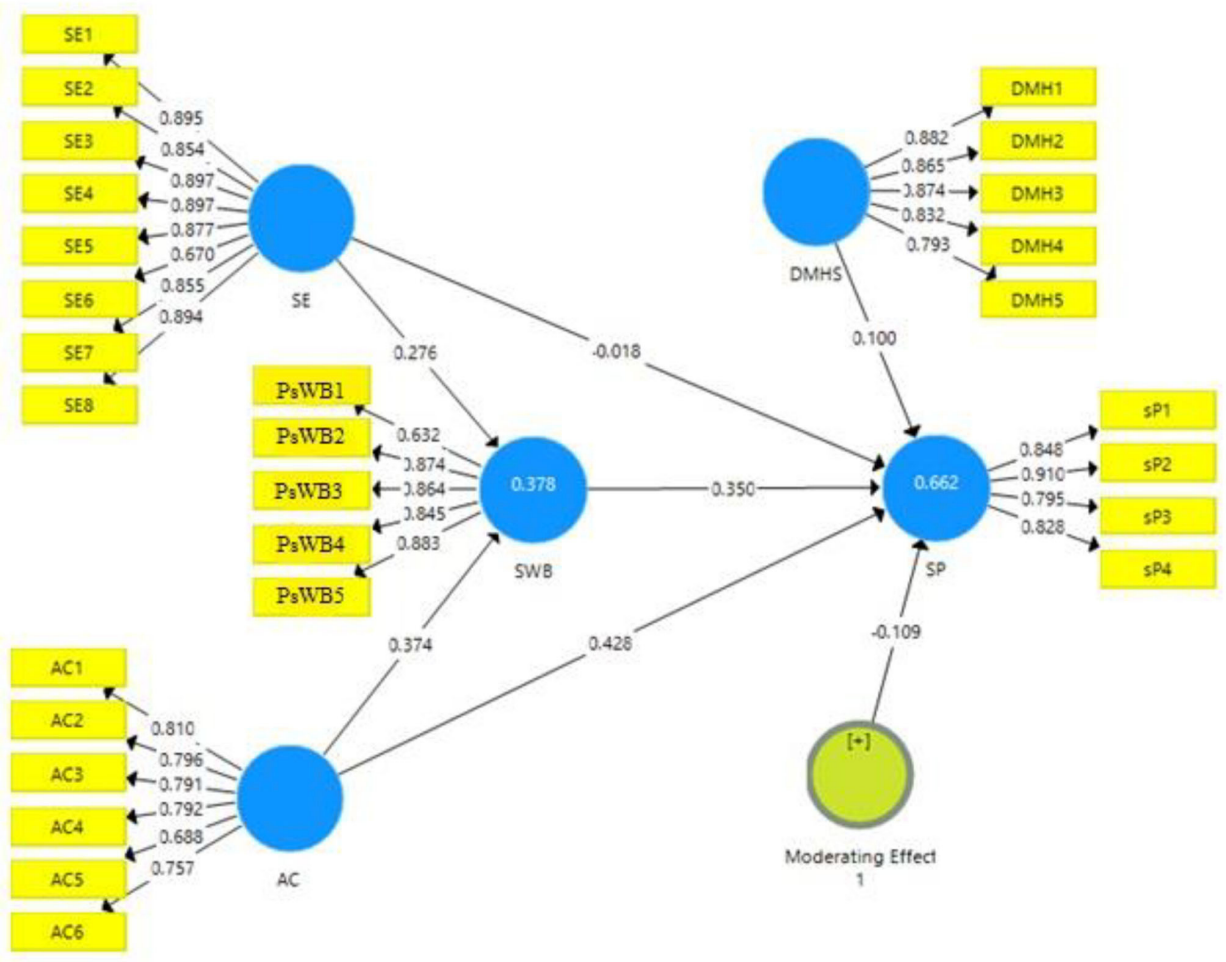
Measurement model. AC, affective commitment; DMHS, digital mental health services; SE, self-efficacy; SP, student performance; PsWB, psychological wellbeing.

Figure 2 shows the output of the measurement model algorithm. This model depicts how many independent variables, i.e., SE and affective commitment, contribute to the study's dependent variables, i.e., student performance.

Table 2 shows factor loadings for each item of the variable, i.e., SE, affective commitment, psychological wellbeing, student performance and the digital mental health services, and the variables' Cronbach alpha values, composite reliability, and AVE. Measuring a variable requires multiple items, and each item's contribution toward a construct is determined through factor loadings. According to Jordan et al. (55), the value of factor loading <0.60 is regarded as undesirable, and the value of factor loading >0.60 is regarded as fair. In contrast, a factor loading value >0.70 is regarded as highly desirable. Table 2 shows that factor loadings for each variable item are >0.60; therefore, the factor loadings are fair for all the study variables.

**Table 2:** Factor loadings, Cronbach Alpha, composite reliability, and AVE.

**Variables**	**Factor loadings**		**Cronbach alpha**	**Composite reliability**	**AVE**
Affective commitment	AC1	0.810	0.865	0.899	0.598
	AC2	0.796			
	AC3	0.791			
	AC4	0.792			
	AC5	0.688			
	AC6	0.757			
Digital mental health	DMH1	0.882	0.904	0.929	0.722
	DMH2	0.865			
	DMH3	0.874			
	DMH4	0.832			
	DMH5	0.793			
Self-efficacy	SE1	0.895	0.917	0.927	0.726
	SE2	0.854			
	SE3	0.897			
	SE4	0.897			
	SE5	0.877			
	SE6	0.670			
	SE7	0.855			
	SE8	0.894			
Psychological wellbeing	PsWB1	0.632	0.881	0.913	0.681
	PsWB2	0.874			
	PsWB3	0.864			
	PsWB4	0.845			
	PsWB5	0.883			
Students' performance	sP1	0.848	0.868	0.910	0.716
	sP2	0.910			
	sP3	0.795			
	sP4	0.828			

Table 2 shows the Cronbach alpha for each study variable. The result indicates that internal consistency between the items of the variables exists because the values are higher than 0.70 (56). Moreover, according to Peterson and Kim, (57) the values of composite reliability have three different ranges, the value equal to 0.60 is considered fair, between 0.60 and 0.70 is considered satisfactory, and between 0.70 and 0.90 is considered highly satisfactory. Table 2 indicates that composite reliability for the variables is satisfactory as the values are more than 0.60. The average variance extracted (AVE) for the four variables is more than 0.50. However, the AVE for one variable is less than 0.50, indicating that convergent validity is not present in that variable (58).

The variables of a study must be different from one another to carry out a study. This difference is examined by the discriminate validity of a variable which is analyzed through the HTMT ratio and Fornell and Larker Criteria. A value below 0.90 signifies that discriminant validity exists between the variables using the HTMT ratio (59). Table 3 shows the result for the discriminant validity of the variables using the HTMT ratio. It can be seen that the values are below 0.90; therefore, discriminant validity exists.

**Table 3:** HTMT ratio.

	**AC**	**DMHS**	**SE**	**SP**	**SWB**
AC					
DMHS	0.597				
SE	0.830	0.474			
SP	0.829	0.593	0.679		
PsWB	0.659	0.587	0.611	0.776	

*AC, affective commitment; DMHS, digital mental health services; SE, self-efficacy; SP, student performance; PsWB, psychological wellbeing*.

Fornell and Larker Criterion are also used to examine whether the variables are different from each other or not. According to Fornell and Larcker (60), the first value of each column should be higher than the following values. The result of the Fornell and Larker Criterion for the present study (see Table 4) shows that discriminant validity is present as the first value of each column should be higher than the following values.

**Table 4:** Fronell and Larcker criteria.

	**AC**	**DMHS**	**SE**	**SP**	**SWB**
AC	0.773				
DMHS	0.529	0.850			
SE	0.756	0.442	0.858		
SP	0.730	0.541	0.626	0.846	
PsWB	0.591	0.531	0.570	0.699	0.825

*AC, affective commitment; DMHS, digital mental health services; SE, self-efficacy; SP, student performance; PsWB, psychological wellbeing*.

The *r*^2^ value of the independent variable i.e., mental health and the values for the dependent variables i.e., student performance and psychological wellbeing. *R*^2^ for psychological wellbeing is shown as 37.4%, meaning that 37.4% of the data fit the regression model. *R*^2^ for student performance is shown as 65.6%, meaning that 65.6% of the data fit the regression model.

### Structural Model

**Figure 3:**
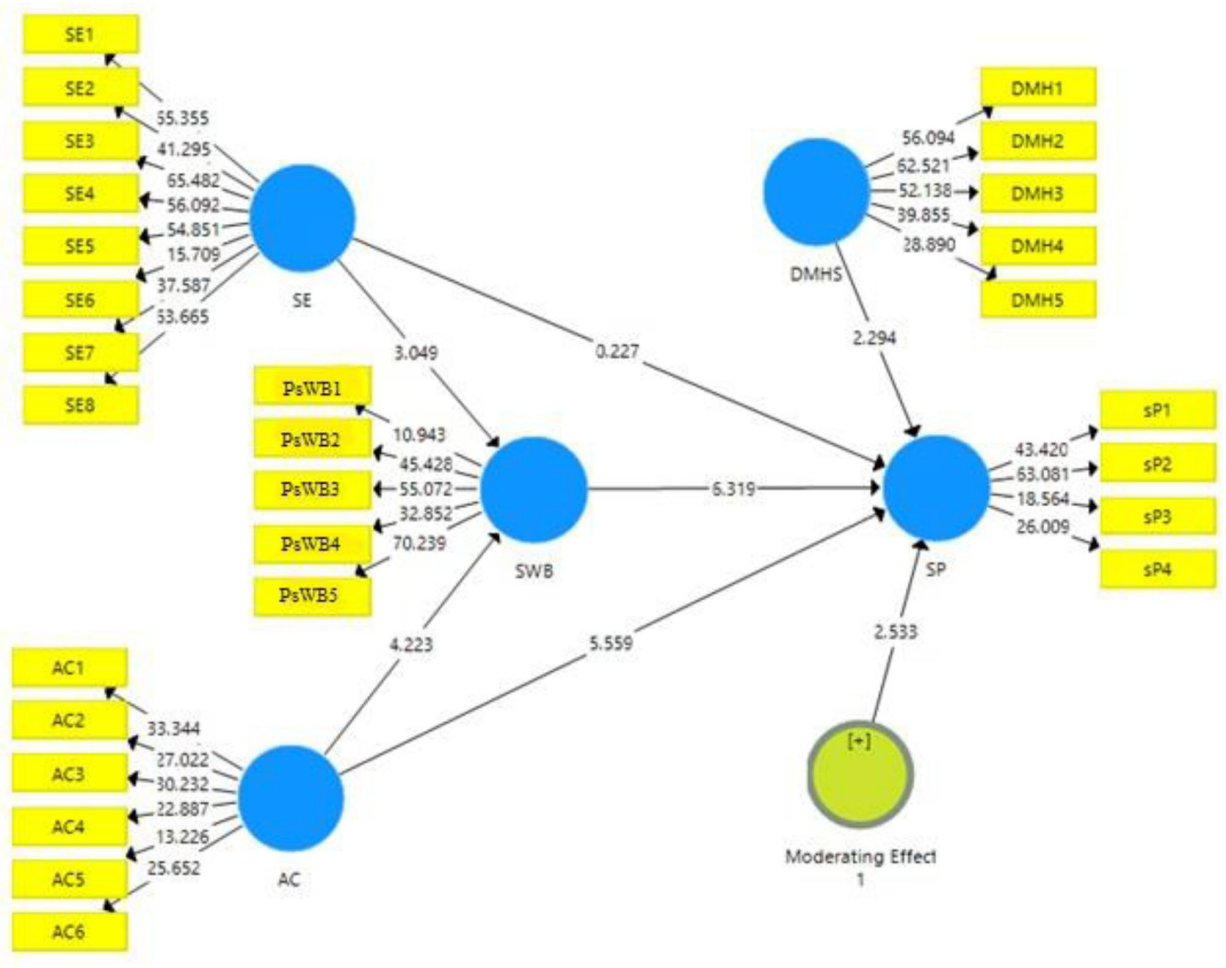
Structural model. AC, affective commitment; DMHS, digital mental health services; SE, self efficacy; SP, student performance; PsWB, psychological wellbeing.

The examination of the structural model is the second stage of data analysis. The relationship between the proposed variables is validated through this model with the help of the PLS-SEM bootstrapping model, which can be seen in Figure 3. The proposed hypotheses of the current study were tested through the result of coefficient, *P*-values, t-statistics, and standard error. A 95% corrected bootstrap has been used to examine the study's relationship.

PLS-SEM bootstrapping was analyzed to test the hypothesis. The bootstrapping was run on the model with moderating effect on organizational climate. The result can be seen in Tables 5, 6 (direct and indirect effects of the variables) and Table 7 (moderating effects of the variables). The study's hypotheses were accepted or rejected based on t-statistics and *P*-values. According to Johnson (61), the value must be greater than 1.96 (*t* > 1.96). The value of *P*-value should be less than 0.05 (*P* < 0.05) (62).

**Table 5:** Direct effects.

**Paths**	**H**	**O**	**M**	**SD**	**T-Statistic**	***P*** **-value**	**Results**
SE → SP	H_1_	−0.018	−0.010	0.078	0.227	0.821	Rejected
AC → SP	H_2_	0.428	0.430	0.077	5.559	0.000	Accepted
PsWB → SP	H_3_	0.350	0.343	0.055	6.319	0.000	Accepted

*AC, affective commitment; DMHS, digital mental health services; SE, self-efficacy; SP, student performance; PsWB, psychological wellbeing*.

**Table 6:** Indirect effects.

**Paths**	**H**	**O**	**M**	**SD**	**T-Statistic**	***P*** **-value**	**Results**
SE → PsWB → SP	H_4_	0.097	0.093	0.035	2.730	0.007	Accepted
AC → PsWB → SP	H_5_	0.131	0.130	0.035	3.686	0.000	Accepted

*AC, affective commitment; DMHS, digital mental health services; SE, self-efficacy; SP, student performance; PsWB, psychological wellbeing*.

**Table 7:** Moderating effects.

**Paths**	**H**	**O**	**M**	**SD**	**T-Statistic**	***P*** **-value**	**Results**
DMHS^*^SE → SP	H_6_	−0.109	−0.109	0.043	2.533	0.012	Accepted

*DMHS, digital mental health services; SE, self-efficacy; SP, student performance*.

* *Relationship between variables*.

Table 5 shows the direct effects of SE and affective commitment on student performance. The first hypothesis of the study postulated that SE has a positive impact on student performance. H_1_ was rejected as (*t* = 0.227; *P* = 0.821). The second hypothesis of the study postulated that affective commitment has a positive impact on student performance. H_2_ was accepted as (*t* = 5.559; *P* < 0.05). The third hypothesis of the study postulated that psychological wellbeing has a positive impact on student performance. H_3_ was accepted as (*t* = 6.319; *P* < 0.05).

Table 6 shows the indirect effects of the study. The fourth hypothesis of the study postulated that psychological wellbeing mediates the relationship between SE and student performance. H_4_ was accepted as (*t* = 2.370; *P* < 0.05). The fifth hypothesis of the study postulated that psychological wellbeing mediates the relationship between affective commitment and student performance. H_5_ was accepted (*t* = 3.686; *P* < 0.05).

Table 7 shows the moderating effect of digital mental health services on the relationship between SE and student performance. The sixth hypothesis of the study postulated that digital mental health services moderate the relationship between SE and student performance. H_6_ was accepted as (*t* = 2.533; *P* < 0.05). The results show that digital mental health services weaken the relationship between SE and student performance.

## Discussion

This research sought to contribute to understanding some mediating and moderating factors in direct relationships between SE, affective commitment, and students' performance. For this purpose, the authors tried to investigate the direct associations of SE and affective commitment with students' performance. The mediating role of PsWB was also tested in the study. The current study also assessed the moderating role of digital mental health services. There is a perception that any person having a sense of personal capacity tends to have improved performance. This kind of performance is expected in every kind of setup, whether it be a professional or educational one. Based on Social Cognitive Theory by Bandura (16), this notion gets enormous support which states that any person having self-confidence in his own abilities is more directed toward better performance.

In the current study, the impact of SE was checked for impacting the students' performance. It is assumed that students are also like the employees of an organization. Therefore, this kind of relationship was studied in this research based on the analogy of employees' SE on their performance. It was assumed that students with strong SE may improve their devotion, effort, and perseverance, resulting in an extraordinary performance. Students with high SE may attribute their failures to a lack of effort rather than a lack of ability, while students with low SE may attribute their failures to a lack of ability (17). The results contradicted this perspective as SE could not develop a directed association with students' performance. This may happen as students are not the paid employees of an organization, rather, they get education on their own by spending money on education.

This may happen as there is a lesser sense of obligation toward them. It may also happen like this due to the fact that students require guidance and training from their mentors and parents to develop a sense of SE. So, SE cannot be developed on its own in the students. It might require a mediator to work significantly. Previously, in contradiction to current research, many researchers found out that SE played an important role in improving employees' performance, e.g., (6, 12, 16, 17, 26). As discussed earlier in the literature review, commitment at the organizational level is of different types, including affective, procedural, and continuance commitments. These types are linked to each other but are separate in function.

It was previously noted that students who had an affective commitment to their institutions were emotionally connected to their educational institutions (28, 29). On the other hand, it was also observed that employees of a certain organization were committed to their organization in a normative way, the second type of organizational commitment. In this kind of commitment, employees consider themselves obliged to the organization. The current study's results suggested that students with affective commitment were emotionally attached to their institutions. Therefore, they performed better due to their emotional attachment. Affective commitment previously showed similar kinds of effects on employees' performance in different research works e.g., (31, 38).

The direct association of PsWB with students' performance was also expected to be positively significant. The results indicated that PsWB significantly influences the students' performance in the current research. This is due to the fact that students' psychological wellbeing is directly associated with the level of happiness and mental health. Previously, it was noted that PsWB can be measured through different variables, including satisfaction, happiness, and overall mental and psychological health. It was also observed that PsWB was linked to both of its types i.e., Hedonic and Eudaimonic (36, 37). The one which played its role in the current research was Eudaimonic which was more related to students as it has an impact on a personal level. The results are also supported by some researchers who evaluated the impact of PsWB on students' performance (38).

The current research also evaluated the indirect mediating and moderating effects of the association of SE, affective commitment, and students' performance. The results indicated that PsWB significantly mediated the relationships between SE, affective commitment, and students' performance. This is due to the fact that the direct association of SE with students' performance in the current research was not significant and needed the help of any facilitating mediators. PsWB positively aided this relationship by providing a mediating link between both. This kind of role of mediator between SE and students' performance was also suggested previously by (10, 34). The digital mental health service also moderated the association for students' performance. As discussed earlier, mental health is quite necessary for performing various activities at educational institutions (44).

Digital mental health services, also referred to as online mental health, e-mental health, telehealth, digital therapy, and web therapy, have been suggested as potential remedies to the mental health care difference among university students and are conveyed *via* virtual machines, mobile phone, or other digital equipment (63). Digital mental health systems can be readily available, less expensive, stigma-reducing, versatile in planning, personalized, and rapid treatment adherence and engagement monitoring (64). Students with mental health issues have shown a generally positive attitude toward online mental health services, with similar numbers expressing potential readiness to seek help online or through campus health services (65). Similar results were also reported previously in which digital mental services provided a helping hand in fighting several depression and anxiety-related issues of the employees and students (15, 44, 45, 47). So, it proved that digital mental health services might regulate performance functioning at any level.

### Theoretical Contribution and Practical Implications

The first and foremost, theoretical contribution of the study is examining the moderating role of digital mental health services on the relationship between SE and student performance. Furthermore, the present study offers a comprehensive model for measuring a thorough relationship between SE with the affective commitment of the students studying in the universities. The present study has found that the higher the affective commitment of the students, the better will be the students' psychological wellbeing, which significantly contributes to their performance. Some of the practical implications of the study are as follows. First, the management of the educational institutions must show concern for the students' mental health by offering them regular sessions on mindfulness training and providing mediation opportunities for the students.

This would enhance the overall psychological wellbeing of the students in their studies. Second, the public sector universities should promote a culture of promoting and providing such a favorable environment that flourishes the SE and affective commitment of the students toward their studies and the institutions. Also, SE can be promoted by allowing the students and giving them opportunities to participate in decision-making assignments and workshops that would give them confidence and the urge to perform better in their studies and extracurricular activities. This act would lead to higher performance and satisfaction among the students. Third, the institutes and universities must be careful about digital mental health services as it has been found to weaken the relationship between SE and student performance.

### Limitations and Future Directions

One of the limitations of the study is related to the target population. This study only included the students at public sector universities in China; therefore, the future study can be conducted on employees of multinational firms or the private sector. Another limitation of the study is the small sample size which might affect the generalizability of the study. Moreover, the study was conducted in China, which could be a limitation of the present study. Thus, the future study can include other regions or other Asian countries to examine those participants' results. The current study took affective commitment as a whole construct; therefore, future studies can examine its related commitments like normative and continuance commitment to understand the model in depth. Furthermore, new variables, such as different leadership styles, can also be introduced in the model to examine their effect on student performance.

## Conclusion

Boost in technology in recent years, especially after the pandemic, has changed the entire realm of the activities and processes carried out in daily life. It has major affected the students' learning style and environment, compelling them to show more commitment and motivation toward their performance. This has also changed the way people seek mental health services to digital services obtained remotely, even at home. For this purpose, the services can be accessed anytime, saving time, energy, and psychological wellbeing. The present study has measured the impact of SE and affective commitment on student performance. Furthermore, digital mental health services' moderating role in the relationship between SE and student performance has also been measured.

The study was conducted on the students currently registered in the public sector universities in China. Hence, the study showed very interesting results for the hypotheses developed. Results showed that affective commitment was positively related to psychological wellbeing and student performance. Also, psychological wellbeing was positively related to the performance of the students, but SE had an insignificant relationship with student performance. Moreover, psychological wellbeing has significantly mediated the relationship between SE, affective commitment, and student performance. Furthermore, digital mental health services were found to weaken the relationship between SE and student performance. Also, in future studies, some new variables can be added to the existing model.

## Data Availability Statement

The original contributions presented in the study are included in the article/supplementary material, further inquiries can be directed to the corresponding author/s.

## Author Contributions

LF: conceived and designed the concept. WM: wrote the article. GJ: supervise the work. All authors read and agreed to the published version of the manuscript.

## Funding

This article is a research project of philosophy and social science planning in Zhejiang Province, which is Further research on the eight-eight strategy - further developing the advantages of the industry with mass characteristics; National Social Science Foundation Project Research on Rural Cultural Construction and all-round development of farmers (project number: 17BKS005); Zhejiang A&F University Research Development Project Research on construction of development-oriented Student Work Model under the New Development Concept (number: 2019FR058); and Phased research results of Zhejiang A&F University Research Development Project General Secretary Xi Jinping's important exposition on ideological and political work in colleges and universities (number: 202360000201).

## Conflict of Interest

The authors declare that the research was conducted in the absence of any commercial or financial relationships that could be construed as a potential conflict of interest.

## Publisher's Note

All claims expressed in this article are solely those of the authors and do not necessarily represent those of their affiliated organizations, or those of the publisher, the editors and the reviewers. Any product that may be evaluated in this article, or claim that may be made by its manufacturer, is not guaranteed or endorsed by the publisher.
